# 

**Published:** 1894-11

**Authors:** 


VOL. XV.] PUBLISHED MONTHLY AT THREE DOLLARS A YEAR. SINGLE COPIES, THIRTY CENTS. [No. 6.

Copyright, 1893, by W. A. Conklin and R. S. Huidekoper.
Printed for the Editors by the E. SCOTT Co.

### THE JOURNAL
OF
### COMPARATIVE MEDICINE
AND
### VETERINARY ARCHIVES.

PHILADELPHIA.

EDTED AND PUB LSHED BY

RUSH SHIPPEN HUIDEKOPER, M. D., Veterinarian, (Alfort),
W. A. CONKLIN, Ph.D., D. V. S.
W. HORACE HOSKINS, D. V. S. A. W. CLEMENT, D. V. S.

NOVEMBER, 1894.

All communications and payments should be addressed to

Dr. W. HORACE HOSKINS, 3452 Ludlow Street, Philadelphia, Pa.

Copies may be had in London of BAILLIERE, TINDALL & COX.

